# Effect of cuff inflation with lidocaine, saline, and air on tracheal tube cuff pressure during laparoscopic resection of colorectal neoplasms: a randomized clinical trial

**DOI:** 10.1186/s12871-024-02606-6

**Published:** 2024-07-01

**Authors:** Xuan Wang, Jie Zhang, Guangli Zhu, Shenquan Cai, Qingtong Zhang, Manlin Duan, Shanwu Feng

**Affiliations:** 1 Department of Anesthesiology, Women’s Hospital of Nanjing Medical University, Nanjing Women and Children’s HealthCare Hospital, No.123 Tianfei Lane, Mochou Road, Nanjing, 210011, Jiangsu Province P.R. China; 2 Department of Anesthesiology, Affiliated Jinling Hospital, Medical School, Nanjing University, Nanjing, Jiangsu Province P.R. China; 3 Department of Anesthesiology, Li Huili Hospital Affiliated to Ningbo University, Ningbo, Zhejiang Province P.R. China; 4 Department of Anesthesiology, Nanjing BenQ Medical Center, The Affiliated BenQ Hospital of Nanjing Medical University, No. 71 Hexi Avenue, Jianye District, Nanjing, Jiangsu Province 210019 P.R. China

**Keywords:** Endotracheal tube cuff pressure, Laparoscopic surgery, Normal saline, Lidocaine, Tracheal mucosa injury

## Abstract

**Background:**

Tracheal tube cuff pressure will increase after pneumoperitoneum when the cuff is inflated with air, high pressure can cause tracheal mucosal damage. This prospective trial aimed to assess if inflating with normal saline or lidocaine can prevent increase of tracheal tube cuff pressure and tracheal mucosal damage in laparoscopic surgeries with general anesthesia. Whether changes of tracheal tube cuff transverse diameter (CD) can predict changes of tracheal tube cuff pressure.

**Methods:**

Ninety patients scheduled for laparoscopic resection of colorectal neoplasms under general anesthesia were randomly assigned to groups air (A), saline (S) or lidocaine (L). Endotracheal tube cuff was inflated with room-temperature air in group A (*n* = 30), normal saline in group S (*n* = 30), 2% lidocaine hydrochloride injection in group L (*n* = 30). After intubation, tracheal tube cuff pressure was monitored by a calibrated pressure transducers, cuff pressure was adjusted to 25 cmH_2_O (T_0.5_). Tracheal tube cuff pressure at 15 min after pneumoperitoneum (T_1_) and 15 min after exsufflation (T_2_) were accessed. CD were measured by ultrasound at T_0.5_ and T_1_, the ability of ΔCD (T_1-0.5_) to predict cuff pressure was accessed. Tracheal mucous injury at the end of surgery were also recorded.

**Results:**

Tracheal tube cuff pressure had no significant difference among the three groups at T_1_ and T_2_. ΔCD had prediction value (AUC: 0.92 [95% CI: 0.81–1.02]; sensitivity: 0.99; specificity: 0.82) for cuff pressure. Tracheal mucous injury at the end of surgery were 0 (0, 1.0) in group A, 0 (0, 1.0) in group S, 0 (0, 0) in group L (*p* = 0.02, group L was lower than group A and S, *p* = 0.03 and *p* = 0.04).

**Conclusions:**

Compared to inflation with air, normal saline and 2% lidocaine cannot ameliorate the increase of tracheal tube cuff pressure during the pneumoperitoneum period under general anesthesia, but lidocaine can decrease postoperative tracheal mucosa injury. ΔCD measured by ultrasound is a predictor for changes of tracheal tube cuff pressure.

**Trial registration:**

Chinese Clinical Trial Registry, identifier: ChiCTR2100054089, Date: 08/12/2021.

**Supplementary Information:**

The online version contains supplementary material available at 10.1186/s12871-024-02606-6.

## Introduction

Maintaining the endotracheal tube cuff pressure within 20–30 cmH_2_O is a standard practice during general anesthesia with orotracheal intubation. Cuff pressure less than 20 cmH_2_O can not provide a tight seal to prevent aspiration while more than 30 cmH_2_O can cause tracheal mucosal damage and airway complications such as sore throat, cough, dysphonia, etc [1, 2]. In laparoscopic surgeries, carbon dioxide pneumoperitoneum and Trendelenburg position are employed, these will lead to a displacement of diaphragm and a decrease of intrathoracic volume, then lung compliance will decreased [3, 4]. Numerous studies have found that using normal saline (NS) or lidocaine to inflate the tracheal tube cuff can reduce the incidence of high cuff pressure during anesthesia as well as early and delayed postoperative airway complications in general anesthesia with nitrous oxide [5–7]. However, whether inflating the tracheal tube cuff with NS or lidocaine can alleviate the increase of tracheal tube cuff pressure during total intravenous anesthesia (TIVA) with orotracheal intubation in laparoscopic surgeries has not been studied.

In practice, anesthesiologists barely manage tracheal tube cuff pressure. There are several reasons for this, including the lack of a cuff pressure gauge, the risk of cross-infection because it will be used on multiple patients, and the lack of calibration maintenance [8]. Nowadays, ultrasound is simple to obtain, and it is unknown whether tracheal tube cuff pressure correlates with tracheal transverse diameter (TD) and tracheal tube cuff transverse diameter (CD).

This prospective randomized controlled trial sought to assess endotracheal tube cuff pressure after pneumoperitoneum, the severity of tracheal mucosal damage and postoperative airway complications when the tracheal tube cuff was inflated with room-temperature air, 2% lidocaine or NS with propofol/remifentanil TIVA and to investigate if TD and CD measured by ultrasound can be used to predict tracheal tube cuff pressure.

## Methods

### Ethics

The protocol was approved by the Research Ethics Committee of Jinling Hospital, Jinling School of Clinical Medicine, Nanjing Medical University (Ethical Application Reference: 2022DZKY-024-01 Nanjing, China) on 18 March 2022. All methods were performed in accordance with relevant guidelines and regulations with CONSORT recommendations [9]. Written informed consent was obtained from all subjects participating in the trial. The trial was registered prior to patient enrollment at the Chinese Clinical Trial Registry (Identifier: ChiCTR2100054089, URL: https://www.chictr.org.cn/edit.aspx?pid=142785&htm=4, Principal investigator: Manlin Duan, Date: 08/12/2021).

### Study design and population

This prospective, randomized, single-center controlled trial included 90 patients aged 18–65 years with American Society of Anesthesiologists (ASA) physical status I ~ III who underwent laparoscopic resection of colorectal neoplasms under TIVA and orotracheal intubation between October 2022 and July 2023. Patients who were unable to provide informed consent; had BMI < 19 kg m^− 2^ or > 30 kg m^− 2^; had Mallampati classification III or IV; had preexisting sore throat, hoarseness, cough, or hemorrhage of laryngeal mucosa; were intubated more than once during anesthesia induction; experienced bucking during surgery; had delayed emergence (inability to regain an adequate level of consciousness, unresponsiveness, or deep sedation for over 60 min from the last administration of the anesthetic agents [10, 11]); required reintubation after extubation within 48 h; had asthma, chronic obstructive pulmonary disease; psychiatric disorders; and were smokers were excluded.

### Randomization

The online computer system ‘OPEN-randomize’ was used to randomly assign patients to the groups, and it generated the randomization sequence to ensure equal distribution [12]. No restrictions applied for random selection, and the numbers for allocation were packaged in opaque envelopes, which could be observed by the anesthesiologist, who has more than 5 years of experience and conducted intubation and extubation. Patients, outcome evaluators, data information analysts were blinded to the trial intervention.

Endotracheal tube cuff inflation was performed using room-temperature air on patients in the air (A) group (*n* = 30), NS in the saline (S) group (*n* = 30), and 2% lidocaine hydrochloride injection in the lidocaine (L) group (*n* = 30) (5 ml: 0.1 g, H142021839, Tiansheng Pharmaceutical CO., LTD. Hubei, China).

### Anesthesia protocol and inflation

All the patients underwent standardized monitoring procedures after entering the operation room: electrocardiography, the saturation of haemoglobin with oxygen, invasive blood pressure and bispectral index (BIS) monitoring. After preoxygenation with 100% oxygen (O_2_), anesthesia was induced using sufentanil 0.4 µg kg^− 1^, propofol 2.0 mg kg^− 1^, and cisatracurium 0.2 mg kg^− 1^. An endotracheal tube reinforced with steel and a tapered cuff (cuffed, Hisern Medical, Zhejiang, China) was used. The tube size was determined by the inner diameter: 7.0–7.5 mm was used for women and 7.5–8.0 mm for men. The patients were intubated within 30 s by the anesthesiologist using GlideScope (ANSHIDA, YL01-II, Jiangsu, China). The anesthesiologist randomly assigned the endotracheal tube cuff to be continuously and slowly inflated with different substances using the minimum air leakage method, and the depth of the tracheal tube was determined by feeling the tracheal tube cuff when pressure was applied at the level of the suprasternal notch [13]. Then cuff pilot was connected to calibrated pressure transducers (Hisern Medical; Zhejiang, China). The transducers were primed with NS, connected to an anesthesia monitor (BeneView T6; Mindray, Shenzhen, China), and zeroed at the level of the patient’s trachea, at the suprasternal notch. The pressure transducer was positioned and attached to the surgical table at the level of the trachea, proximal to the patient’s neck. Subsequently, the tracheal tube cuff pressure was adjusted to a baseline value of 25 cmH_2_O with zero end-expiratory pressure during a brief period of apnea. The tracheal tube cuff was then placed under the green drapes. During surgeries, the substance within the endotracheal tube cuff was not adjusted unless a major leakage occured or the cuff pressure was more than 50 cmH_2_O. The endotracheal tube cuff pressure was continuously monitored until the end of surgery. The patients were covered with an upper-body warming blanket (Bair Hugger, 3 M, St. Paul, MN), and the temperature was set to 38 °C. The room temperature was maintained at 24 °C. All surgeries were performed by the same surgeon. At the beginning of the surgery, patients were placed in a neutral position. After peritoneal insufflation, patients were placed in the 15° head-down position and the surgeon determined the peritoneal insufflation pressure. At the end of surgery, all patients returned to the neutral position.

Anesthesia was maintained using remifentanil 0.5 µg kg^− 1^ min ^− 1^, propofol 6–8 mg kg^− 1^ h^− 1^, and cisatracurium 0.05 mg kg^− 1^ administered every 30 min; an additional dose of cisatracurium was added if necessary. The patients underwent deep neuromuscular blockade during ventilation, and a train of four (TOF) of zero was maintained. Intraoperative mechanical ventilation was determined according to the institution protocol (volume-controlled ventilation; initial respiratory rate of 10–12 breaths min^− 1^, I: E ratio of 1:2, positive end-expiratory pressure of 0). The tidal volumes were 6–8 before insufflation and 8 ml kg^− 1^ of the ideal body weight during insufflation, respectively. The BIS value was maintained at 40–60, end-tidal CO_2_ (EtCO_2_) was 35–45 mmHg by adjusting the respiratory rate, and kept blood pressure fluctuation within ± 20%. Before subcuticular wound closure, administration of propofol and remifentanil was ceased, and 0.2 µg kg^− 1^ sufentanil was administered via intravenous injection. Postoperatively, all patients were transferred to the post-anesthesia care unit.

During recovery from general anesthesia, sputum around the airway and endotracheal tube cuff was suctioned. No medications other than neostigmine and atropine were administered to antagonize residual neuromuscular blockade before extubation. Once the patients conformed to the indications of extubation (TOF ≥ 0.9, EtCO_2_ < 45 mmHg on spontaneous respiration, and ability to follow voice command), the endotracheal tube cuff was deflated by the same anesthesiologist, and then the tracheal tube was slowly and gently removed.

All the patients received patient-controlled intravenous analgesia post-operatively: sufentanil 50 µg; dexmedetomidine 200 µg and ondansetron 8 mg diluted to 100 ml with NS. The dose was controlled at 1.5 ml at all times, the rate was 2 ml h ^− 1^, and was locked for 15 min.

### Measurement of TD and CD using ultrasound

**Fig. 1:**
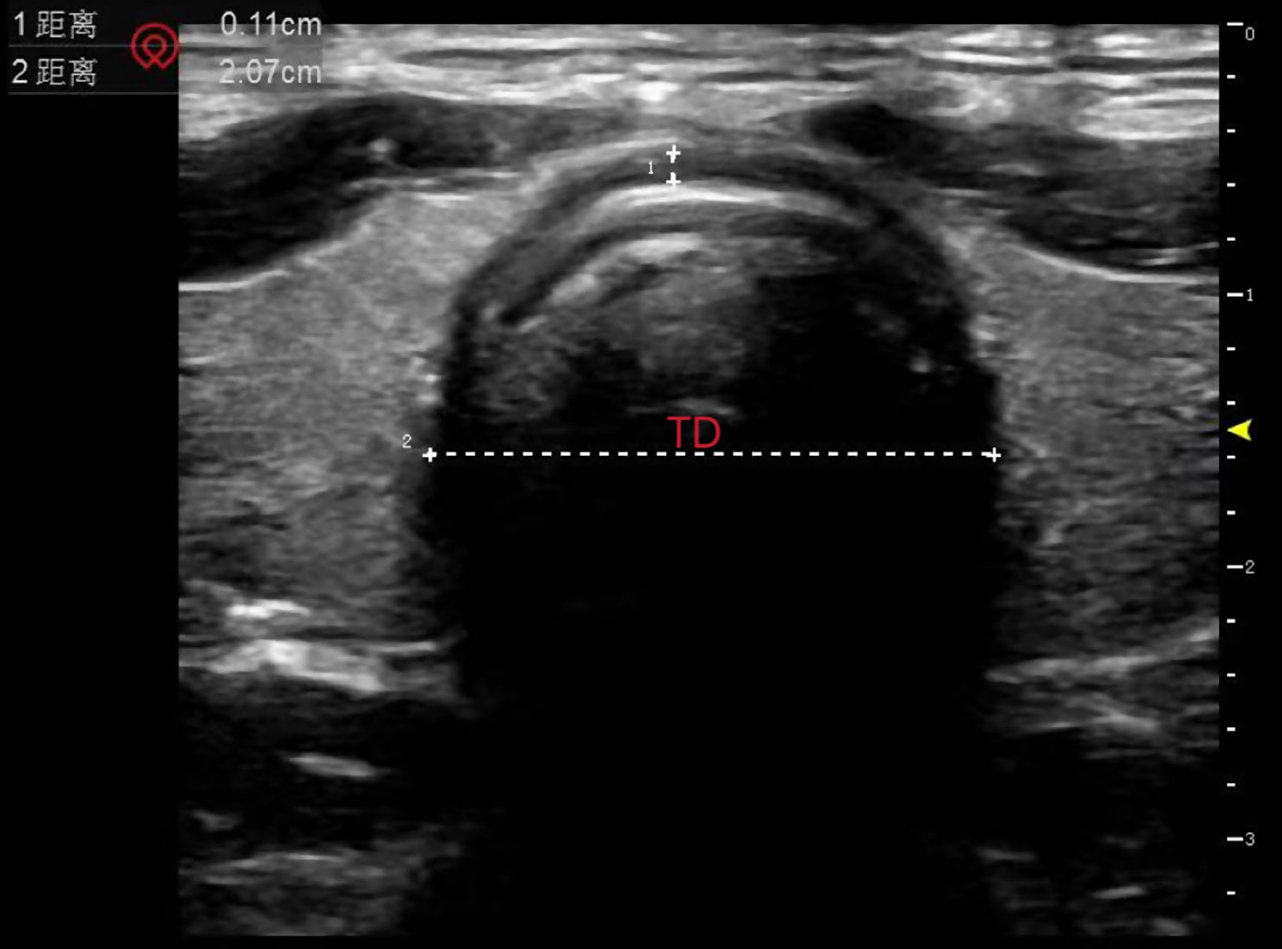
The TD at the level of the suprasternal notch measured by ultrasound. The high-frequency hockey stick probe was coated with ultrasound gel before being held perpendicular to the midline of the trachea and the TD at the level of the suprasternal notch was measured. The shadow: trachea; TD: tracheal transverse diameter

**Fig. 2:**
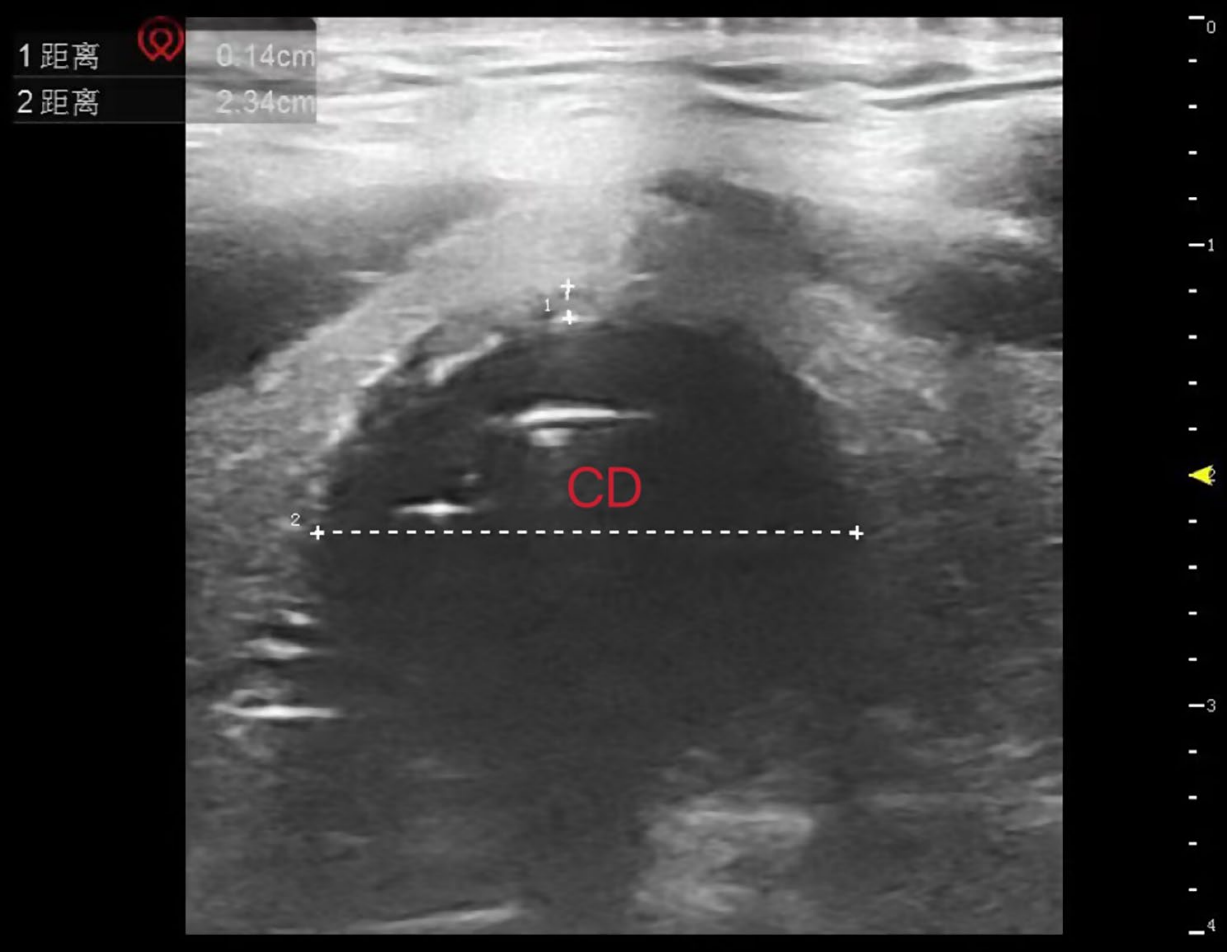
The CD at the level of the suprasternal notch measured by ultrasound. To find the maximal diameter of the tracheal tube cuff, we first placed the probe on the level of the suprasternal notch to confirm the placement of the tracheal tube cuff, then slowly moved the probe upright toward the head to find the maximal diameter of the tracheal tube cuff. The shadow: cuff; CD: tracheal tube cuff transverse diameter

TD and CD were measured when the cuff pressure was adjusted to 25 cmH_2_O and 15 min after pneumoperitoneum. Variation of the two-time points indicated TD’s variation (ΔTD) and CD’s variation (ΔCD). Patients were measured while lying supine with their heads in a neutral position. The head, pharynx, and throat were all in one line. Measurements were done using ultrasound (Navis, Wisonic, Shenzhen, China). The high-frequency hockey stick probe was coated with ultrasound gel before being held perpendicular to the midline of the trachea and the TD at the level of the suprasternal notch was measured (Fig. 1). To find the maximal diameter of the tracheal tube cuff, we first placed the probe on the level of the suprasternal notch to confirm the placement of the tracheal tube cuff, then slowly moved the probe upright toward the head to find the maximal diameter of the tracheal tube cuff (Fig. 2).

### Evaluation of tracheal mucous injury using a bronchofiberscope

**Fig. 3:**
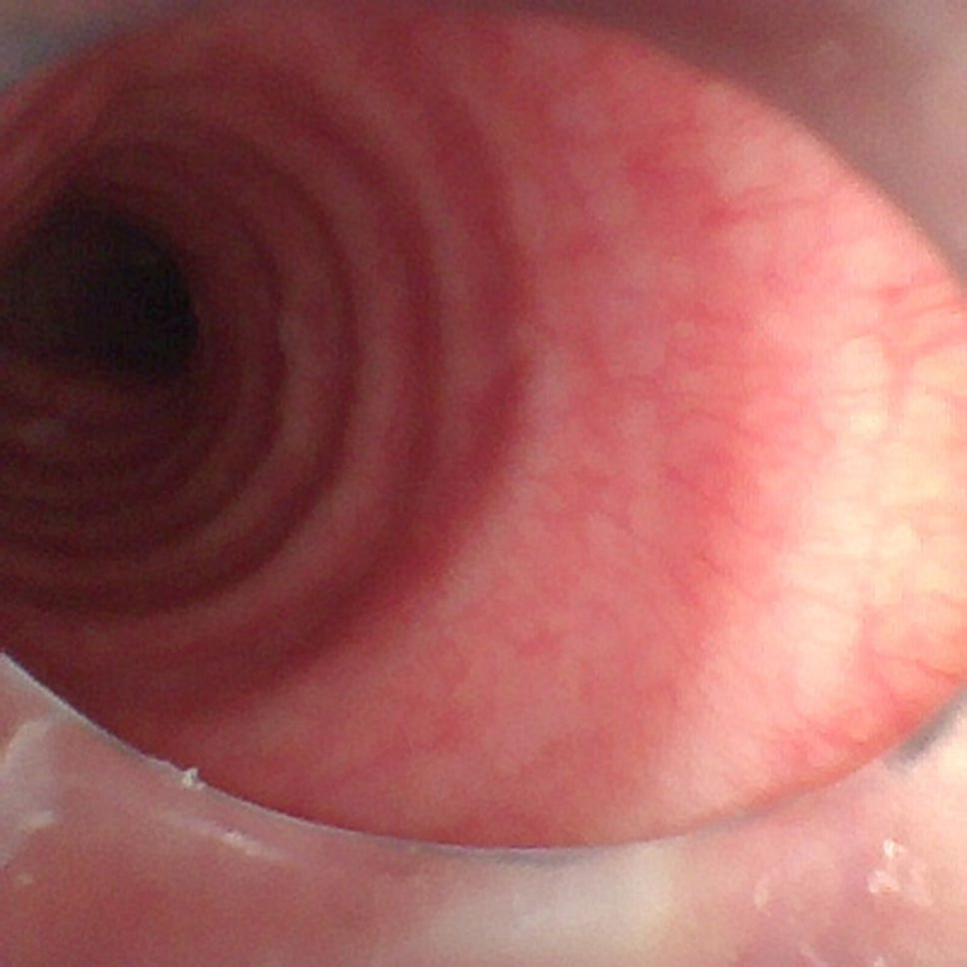
Evaluation of tracheal mucous injury using a bronchofiberscope. The bronchofiberscope was inserted using the transtracheal tube until it reached the tracheal tube cuff. Then, the cuff was deflated, and the tracheal tube was extubated carefully to avoid the tip of the tracheal tube falling into the glottis

We examined the tracheal mucosa with a bronchofiberscope (ANSHIDA, YL05-F, Jiangsu, China) at the end of surgery. The bronchofiberscope was inserted via the tracheal tube until it reached the tracheal tube cuff. Then, the cuff was deflated, and the tracheal tube was extubated carefully to avoid the tip of the tracheal tube beyond the glottis (Fig. 3). The distance between the top of the tracheal tube cuff and the tip of the tracheal tube is about 6.5 cm, so the tube is extubated 7–8 cm to allow for direct evaluation of the mucous where the tracheal tube cuff is located. Then, the tracheal tube was intubated to the original depth along the bronchofiberscope.

### Different time points

The various time points were patients entered the operating room (T_0_); the cuff pressure was adjusted to 25 cmH_2_O (T_0.5_); 15 min after pneumoperitoneum (T_1_); 15 min after exsufflation (T_2_).

### Outcome measures

The primary goal was to compare the tracheal tube cuff pressure at various time points in the three groups. The secondary goal was to determine the relationship between endotracheal tube cuff pressure, ΔTD, and ΔCD; tracheal mucous injury at the end of surgery; the severity of postoperative airway complications such as sore throat, hoarseness, and hemorrhage of the laryngeal mucosa at 2, 24, and 48 h postoperatively. Patient’s demographic information; peak airway pressure, mean airway pressure, respiratory rate from T_0.5_ to T_2_; peritoneal insufflation pressure at T_1_; the durations of surgery, pneumoperitoneum and anesthesia (from anesthesia induction to extubation) were also determined. The tracheal mucous injury was graded by self-made classification accordingly: 0 indicates no injury; 1 indicates congestion or edema; 2 indicates punctate hemorrhage; 3 indicates splinter hemorrhage; 4 indicates ulcer; and 5 indicates fistula. Cough, hoarseness and sore throat were graded on 0–3 scales as follows. Cough: 0 = no cough, 1 = single cough, 2 = more than one episode of unsustained cough, 3 = severe sustained bouts of cough [14]. Hoarseness: 0 = no hoarseness at any time, 1 = no hoarseness in the interview, 2 = hoarseness in the interview noted by the patient only, and 3 = hoarseness are easily noted in the interview. Sore throat: 0 = none, 1 = less severe than with a cold, 2 = similar to that noted with a cold, 3 = more severe than with a cold [15].

### Sample size and statistical analysis

According to our previous study, the incidence of out-of-range of tracheal tube cuff pressure with air inflation is 80%, If a 50% reduction in out-of-range of tracheal tube cuff pressure incidence was determined to be clinically significant, with an effect size of 0.36, degrees of freedom of 2, a power of 80%, and an error of 0.05, ChiSquare test by the Power Analysis and Sample Size Software (version 15.0; NCSS, LLC, USA) calculated that 76 patients were required totally. Given a 20% dropout rate, 96 patients were included, each group was 32 patients. According to the normality of the distribution, continuous study variables were summarized as mean (standard deviation) or median (25-75th percentiles). Frequencies were used to summarize categorical variables (percentage). For normally distributed continuous variables, group differences were tested using one-way analysis of variance (ANOVA). The Levene test for homogeneity of variance was performed, and *p* > 0.1 was considered homogeneous of variance. If the ANOVA result was statistically significant, least signficant difference (LSD) was performed for pairwise comparison when the variance between the groups was homogeneous; otherwise, Dunnett T3 was used. For non-normally distributed continuous variables, the Kruskal–Wallis test was used, and if the result was statistically significant, the Mann–Whitney test was used for pairwise comparison. Categorical variables were compared using the chi-square test. Receiver operating characteristic curve (ROC) analysis was used to assess the prediction ability of ΔTD and ΔCD for tracheal tube cuff pressure. IBM SPSS version 26.0 were used to conduct the statistical analyses. The reported p-values were Bonferroni-corrected. *p* < 0.05 was regarded as statistically significant.

## Results

**Fig. 4:**
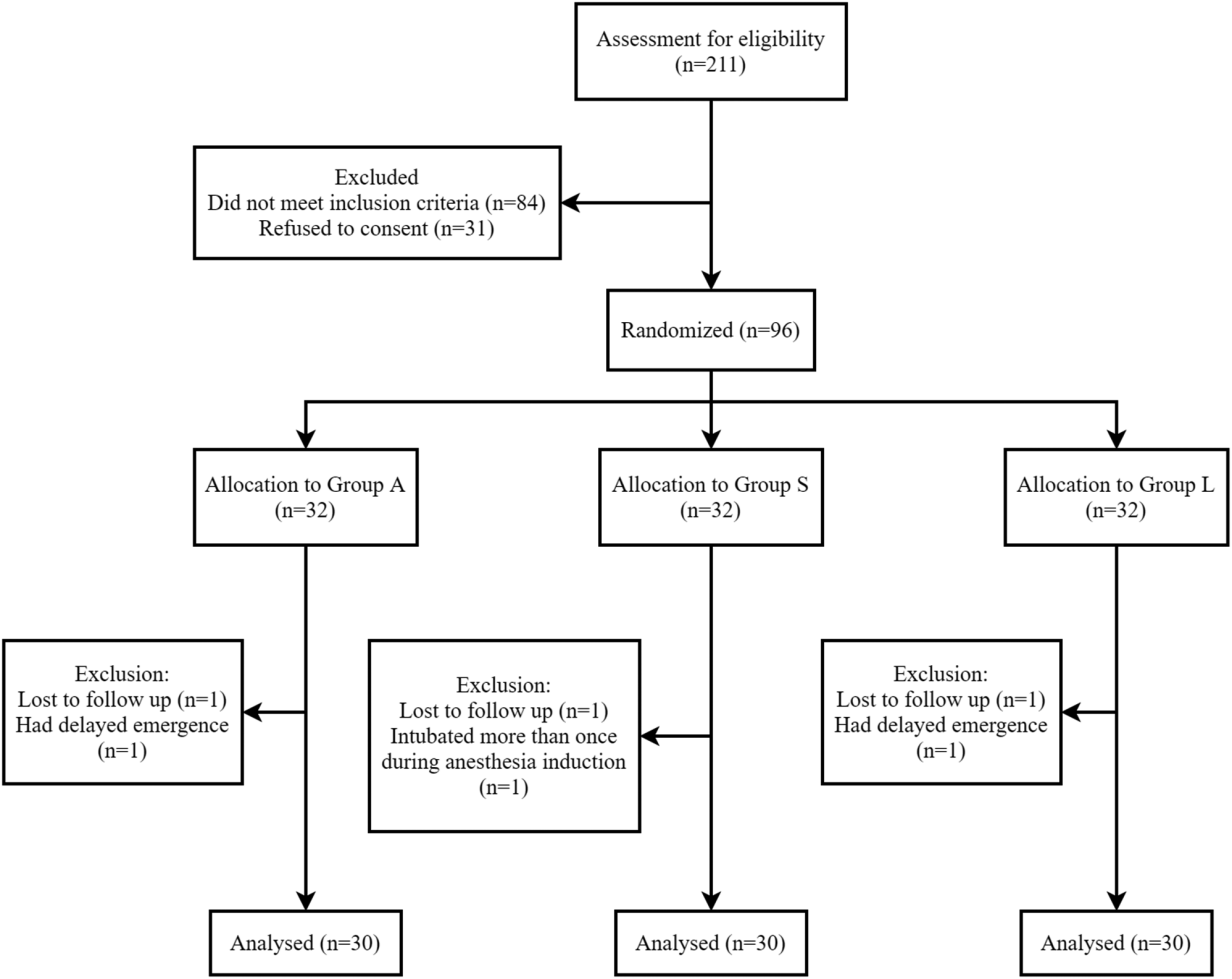
Consort flow chart that outlines patients’ assignment. This study enrolled 96 of 211 eligible patients, and 6 were excluded from the analysis: 3 were lost to follow-up, 1 was intubated more than once during anesthesia induction, and 2 had delayed emergence. Therefore, 90 patients were included in the final analysis

This study enrolled 96 of 211 eligible patients, and 6 were excluded from the analysis: 3 were lost to follow-up, 1 was intubated more than once during anesthesia induction, and 2 had delayed emergence. Therefore, 90 patients were included in the final analysis (Fig. 4).

The characteristics of the patients and the operations were comparable across the groups (Table 1). There were no differences in age, sex, BMI, ASA classification, Mallampati classification among the three groups. There were no significant differences among the groups in terms of the patients’ tracheal tube size, operation duration, anaesthesia duration and pneumoperitoneum duration (Table 1).

**Table 1:** Patients and operation characteristics

	Group A (*n* = 30)	Group S (*n* = 30)	Group L (*n* = 30)
Age (yr)	45.8 (33.3–60.0)	45.5 (33.5–57.5)	46.6 (39.5–55.5)
Female, n(%)	24 (80.0)	18 (60.0)	23 (76.7)
BMI (Kg m^− 2^)	22.3 (19.2–25.6)	23.5 (20.4–26.0)	23.3 (20.7–25.2)
ASA classification, n(%)			
I	5 (16.7)	2 (6.7)	4 (13.3)
II	23 (76.7)	26 (86.7)	25 (83.3)
III	2 (6.7)	2 (6.7)	1 (3.3)
Mallampati classification, n(%)			
I	12 (40.0)	9 (30.0)	16 (53.3)
II	18 (60.0)	21 (70.0)	14 (46.7)
Operation duration (min)	162.1 ± 77.8	162.3 ± 67.1	153.0 ± 44.0
Pneumoperitoneum duration (min)	98.5 ± 53.8	94.0 ± 40.6	88.2 ± 27.4
Anaesthesia duration (min)	223.6 ± 83.0	216.1 ± 77.9	201.5 ± 55.9
Tube size, n(%)			
7.0	24 (80.0)	19 (63.3)	23 (76.7)
7.5	6 (20.0)	11 (36.7)	7 (23.3)

Abbreviations: BMI, Body Mass Index; ASA, American Society of Anesthesiologists. SD, standard deviation. The values are expressed as mean ± SD, median (25-75th percentiles), or number of patients (percentage). *P* < 0.05 is considered statistic significant

The tracheal tube cuff pressure at T_1_ was 33.8 ± 3.6 cmH_2_O in group A, 33.1 ± 3.3 cmH_2_O in group S, and 33.1 ± 4.2 cmH_2_O in group L; there was no significant difference between the three groups (Table 2). Furthermore, the three groups’ tracheal tube cuff pressure at T_2_ was comparable.

**Table 2:** Intraoperative Ventilatory Characteristics

	Group A (*n* = 30)	Group S (*n* = 30)	Group L (*n* = 30)	*P* values
Tracheal tube cuff pressure (cmH_2_O)				
T_1_	33.8 ± 3.6	33.1 ± 3.3	33.1 ± 4.2	0.68
T_2_	27.7 ± 3.8	26.8 ± 3.2	26.0 ± 3.4	0.18
Peak airway pressure (cmH_2_O)				
T_0.5_	16.3 ± 2.7	15.8 ± 2.5	15.6 ± 3.6	0.64
T_1_	25.1 ± 3.5	22.9 ± 4.1	22.8 ± 5.0	0.07
T_2_	16.7 ± 2.9	16.1 ± 2.2	17.0 ± 4.0	0.50
Mean airway pressure (cmH_2_O)				
T_0.5_	6.4 ± 1.1	6.4 ± 1.0	6.1 ± 1.1	0.34
T_1_	8.2 ± 0.9	7.9 ± 1.3	7.9 ± 1.4	0.52
T_2_	6.7 ± 1.0	6.5 ± 0.9	6.6 ± 1.1	0.73
Tidal volume (ml)				
T_0.5_	435.7 ± 56.0	465.0 ± 53.6	452.3 ± 70.8	0.18
T_1_	411.8 ± 48.0	426.3 ± 49.3	425.2 ± 65.0	0.52
T_2_	422.7 ± 56.1	450.5 ± 50.5	444.7 ± 66.2	0.15
Respiratory rate (per minute)				
T_0.5_	12.9 ± 1.1	12.8 ± 1.0	12.8 ± 1.1	0.92
T_1_	14.7 ± 1.0	14.8 ± 0.9	14.5 ± 1.0	0.64
T_2_	13.8 ± 1.1	13.3 ± 1.3	13.2 ± 1.3	0.20
Peritoneal insufflation pressure (mmHg)				
T_1_	11.9 ± 0.9	11.8 ± 0.8	11.9 ± 0.8	0.88

The values are expressed as mean ± SD. *P* < 0.05 is considered statistic significant

T_0_, patients entered the operating room; T_0.5_, the cuff pressure was adjusted to 25 cmH_2_O; T_1_, 15 min after pneumoperitoneum; T_2_, 15 min after exsufflation

Abbreviations: SD, standard deviation

Peak airway pressure, mean airway pressure, respiratory rate, tidal volume, peritoneal insufflation pressure were all comparable among the three groups at different time points respectively (Table 2).

**Fig. 5:**
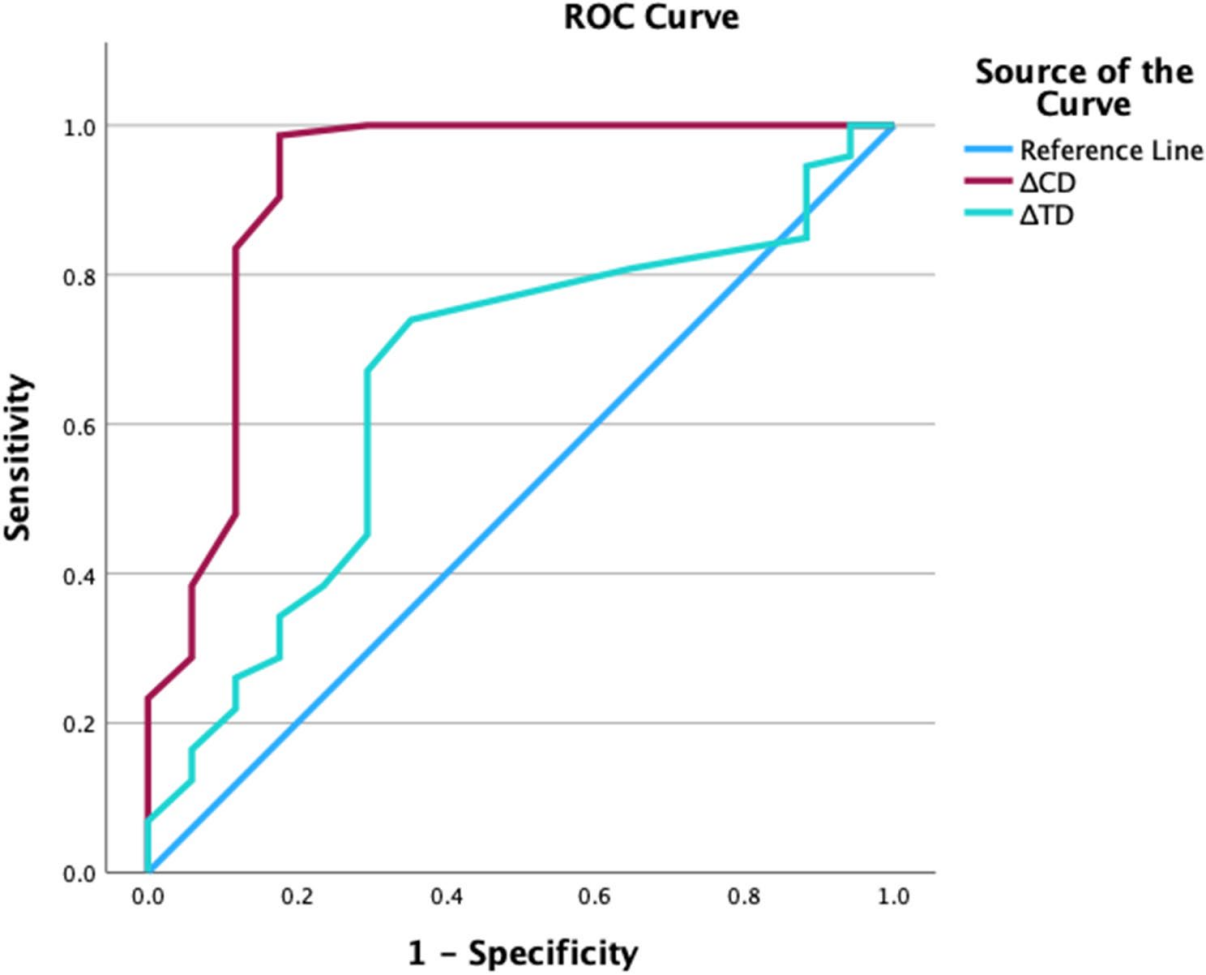
*ROC for* ΔCD and ΔTD for prediction of tracheal tube cuff pressure. ΔCD had the highest prediction value (AUC: 0.92 [95% CI: 0.81–1.02]; cutoff: 0.03; sensitivity: 0.99; specificity: 0.82), ΔTD had no prediction value (AUC: 0.66 [95% CI: 0.51–0.80]; cutoff: 0.05; sensitivity: 0.74; specificity: 0.65). ROC: receiver operating characteristic curve; ΔTD: tracheal transverse diameter’s variation; ΔCD: tracheal tube cuff transverse diameter’s variation; AUC: area under curve; CI: confidence interval

To predict out-of-range tracheal tube cuff pressure, ΔCD had the highest prediction value (area under curve (AUC): 0.92 [95% confidence interval (CI): 0.81–1.02]; cutoff: 0.03; sensitivity: 0.99; specificity: 0.82), while ΔTD had no prediction value (AUC: 0.66 [95% CI: 0.51–0.80]; cutoff: 0.05; sensitivity: 0.74; specificity: 0.65) (Fig. 5, Supplementary Table 1).

The scores of tracheal mucous injury evaluated by bronchofiberscope at the end of surgery were 0 (0–1.0) in group A, 0 (0–1.0) in group S, 0 (0–0) in group L (*p* = 0.02 for all groups, group L was lower than group A and S, *p* = 0.03 and *p* = 0.04, respectively, Table 3).

**Table 3:** Perioperative airway complications

	Group A (*n* = 30)	Group S (*n* = 30)	Group L (*n* = 30)	*P* values
Tracheal mucous injury (score)	0 (0–1.0)^a^	0 (0–1.0)^a^	0 (0–0)	0.02
Postoperative airway complications				
2 h (score)	0.9 (0–2.0)	0.7 (0-1.3)	0.4 (0–1.0)*	0.04
24 h (score)	0.6 (0–1.0)	0.4 (0–1.0)	0.3 (0-0.3)	0.17
48 h (score)	0.3 (0–1.0)	0.3 (0-0.3)	0.2 (0-0.3)	0.74
Incidence of complications at 2 h after surgery, n(%)				
Sore throat	16 (53.3)	12 (40.0)	6 (20.0)*	0.02
Cough	14 (46.7)	18 (60.0)	11 (36.7)	0.19
Others	9 (30.0)	7 (23.3)	8 (26.7)	0.84

The values are expressed as median (25-75th percentiles). ^a^*P* < 0.05 vs. group L, ^*^P < 0.05 vs. group A. *P* < 0.05 is considered statistic significant

The severity of postoperative airway complications at 2 h was graded as 0.9 (0–2.0) in group A, 0.7 (0-1.3) in group S, and 0.4 (0–1.0) in group L (*p* = 0.04 for group comparisons, Table 3), group L was lower than group A (*p* = 0.02, Table 3). The postoperative airway complications were mainly sore throat and cough. The incidence of sore throat in group A was 53.3%, in group S was 40.0% and in group L was 20.0% (*p* = 0.02 for all groups, *p* = 0.02 between group A and L, Table 3). The incidence of cough and other complications were comparable among the three groups (Table 3).

The severity of postoperative airway complications at 24 h was graded as 0.6 (0–1.0) in group A, 0.4 (0–1.0) in group S, and 0.3 (0-0.3) in group L. There was no statistically significant difference between the three groups. The severity of postoperative airway complications at 48 h was graded as 0.3 (0–1.0) in group A, 0.3 (0-0.3) in group S, and 0.2 (0-0.3) in group L. There was no statistically significant difference between the three groups (Table 3).

## Discussion

In this trial, we assumed that lidocaine injection or NS could keep the tracheal tube cuff pressure from exceeding the safe limit during the pneumoperitoneum period. However, we discovered that using NS or 2% lidocaine injection to inflate the tracheal tube cuff did not prevent the increase of tracheal tube cuff pressure. This goes against our expectations. We believe it is due to cephalad displacement of the diaphragm and lung volume reduction during the pneumoperitoneum period, which causes patients’ respiratory compliance to decrease. As previously reported in laparoscopic pelvic surgeries, mechanical ventilation can increase tracheal tube cuff pressures in patients with decreased respiratory compliance [16]. This is why liquids were unable to prevent an increase in tracheal tube cuff pressure in our study.

Various pharmacological strategies were used to prevent postoperative sore throat (POST) and other airway complications, such as opioids, steroids, anti-inflammatory drugs, or local anesthetics [17]. Because of safety and feasibility, lidocaine is one of the most commonly used drugs for preventing POST. In this study, we used 2% lidocaine hydrochloride injection to inflate the tracheal tube cuff and the result showed that inflating with 2% lidocaine injection can reduce tracheal mucosal injury and airway complications at 2 h postoperatively. This is consistent with previous research, both alkalinized and non-alkalinized intracuff lidocaine could prevent and alleviate POST. 40 mg 2% lidocaine hydrochloride injection without alkalinization alleviated postoperative sore throat during the first 2 h [18]. Lidocaine hydrochloride injection placed inside the cuff can slowly diffuse through the hydrophobic structure and got a localized effect on the trachea, resulting in improved trachea tolerance and lower incidence of cough, which would decrease POST [6]. What else, inflation with alkalinized lidocaine injection demonstrated a decrease in POST compared with lidocaine hydrochloride injection. However, plasma lidocaine levels would increase through the cuff when lidocaine was alkalinized both in vivo and in vitro studies [18]. As previously reported, lidocaine gel applied to the cuff or intravenous lidocaine could all alleviate POST. For the plasma lidocaine level, intravenous lidocaine would reach 2–3 µg/ml, topical application would range 0.43–1.5 µg/ml, for cuff inflation, alkalinized lidocaine would not beyond 0.08 µg/ml.^17^ The mechanism of lidocaine preventing POST need further investigation.

Ultrasound is increasingly being used for airway assessment and management. It has the potential to serve as both a diagnostic tool and an imaging guide for a variety of procedures. For example, ultrasound can be used to assess the airway, including identify the difficult airway, identify subglottic stenosis and predict pediatric endotracheal tube size. It can be applied to confirm intubation, including direct assessment with transtracheal visualization, indirect assessment with diaphragmatic movement, the depth of endotracheal tube. Lung sliding and cricothyroid membrane also can be assessed by ultrasound [19]. However, no study had reported whether endotracheal tube cuff pressure changes could be predicted by ultrasound. In our study, we discovered that ΔCD is a valuable indicator of high tracheal tube cuff pressure with high sensitivity and specificity. As ultrasound could assist identifying relevant anatomy in a simple, rapid, and noninvasive manner, this result reminded us ultrasound-guided measurement of ΔCD could be a replacement method for tracheal tube cuff pressure management, particularly where a cuff pressure gauge is not available. Due to visual limitations, we cannot measure the contact area between the tracheal lateral wall and the tracheal tube cuff directly with ultrasound, we hypothesized that CD is related to the contact area. When CD increased, the contact area increased too. As tracheal tube cuff pressure rises, so will ΔCD. In our study, the depth of the tracheal tube was determined by feeling the tracheal tube cuff when pressure was applied at the level of the suprasternal notch, because the tracheal tube cuff itself occupied space, the maximal diameter of the tracheal tube cuff must be searched by moving the probe from the level of the suprasternal notch upright toward the head.

While the TD after pneumoperitoneum was nearly identical to the CD, why ΔTD could not predict the increase in tracheal tube cuff pressure? We hypothesized that this was due to TD being wider than CD when the tracheal tube cuff pressure was 25 cmH_2_O, and thus ΔTD being unable to reflect changes in tracheal tube cuff pressure.

This trial has some benefits. There has been no research into whether NS and lidocaine inflated to the tracheal tube cuff could prevent high tracheal tube cuff pressure during CO_2_ pneumoperitoneum. And we first evaluated ΔTD and ΔCD measured by ultrasound to identify out-of-range tracheal tube cuff pressure. The trial, on the other hand, is not without flaws [20]. First, this was a single-center study, and perhaps a multicenter study would have been preferable to further test our hypothesis. Second, in order to minimize invasive procedures on patients, we evaluated tracheal mucosa injury with a bronchofiberscope at the end of surgery without evaluating at the moment after intubation, and the severity of injury may be affected by pneumoperitoneum duration, which we did not consider. Third, the adverse effects of lidocaine injection were not monitored in this study, and the mechanism of tracheal mucosal injury relieved by lidocaine injection inflated to the tracheal tube cuff warrants further investigation.

Conclusively, when compared to inflation with ordinary temperature air to the tracheal tube cuff, saline, and lidocaine cannot ameliorate the increase of tracheal tube cuff pressure during the pneumoperitoneum period under general anesthesia with TIVA, but inflating with 2% lidocaine can decrease tracheal mucosa injury and postoperative airway complications in the 2nd hour after surgery. ΔCD is a useful predictor of out-of-range tracheal tube cuff pressure and may become a replace method of cuff pressure management.

### Electronic supplementary material

Below is the link to the electronic supplementary material.

Supplementary Material 1

## Data Availability

Data is provided within the manuscript or supplementary information files.
