# Supplementary material for: Effect of cuff inflation with lidocaine, saline, and air on tracheal tube cuff pressure during laparoscopic resection of colorectal neoplasms: a randomized clinical trial
Source: BMC Anesthesiol. 2024 Jul 1;24:216. doi: 10.1186/s12871-024-02606-6 (PMC11218051; doi:10.1186/s12871-024-02606-6)
Supplement: Supplementary file 1 — Supplementary Material 1 [file 12871_2024_2606_MOESM1_ESM.docx]

| Supplementary Table.1 AUC and cut-off points for ΔTD and ΔCD | | | | |
| --- | --- | --- | --- | --- |
|  | AUC (95% CI) | Cutoff | Sensitivity | Specificity |
| ΔTD, cm | 0.66 (0.51-0.80) | 0.05 | 0.74 | 0.65 |
| ΔCD, cm | 0.92 (0.81-1.02) | 0.03 | 0.99 | 0.82 |
| Abbreviations: AUC, area under curve; ΔTD, tracheal transverse diameter’s variation; ΔCD: tracheal tube cuff transverse diameter’s variation. CI: confidence interval. *p* < 0.05 is considered statistic significant. | | | | |
